# A Desired Dose of Doubt: Antibiotic Prophylaxis Prior to Transcatheter Congenital Heart Procedures

**DOI:** 10.1016/j.jacadv.2026.102997

**Published:** 2026-07-10

**Authors:** Jack W. McHugh, Supavit Chesdachai, Larry M. Baddour

**Affiliations:** a Division of Public Health, Infectious Diseases, and Occupational Medicine, Department of Medicine, Mayo Clinic, Rochester, Minnesota, USA; b Department of Cardiovascular Medicine, Mayo Clinic, Rochester, Minnesota, USA

**Keywords:** antibiotics, congenital heart disease, endocarditis, infective endocarditis, peri-procedural, transcatheter

**Figure:**
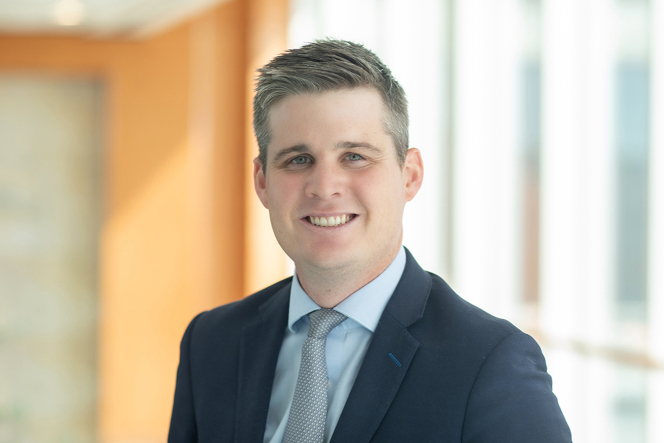

Antibiotic prophylaxis (ABP) to prevent infective endocarditis (IE) before congenital cardiac catheterization sits in a familiar evidentiary gap: the biologic rationale is plausible, the consequences of IE can be severe, and randomized trial data are lacking. In this issue, Issa et al^1^ use retrospective Pediatric Health Information System (PHIS) data to describe variation in periprocedural ABP use and assess its association with IE within 30 days. The question matters because patients with congenital heart disease have an elevated lifetime IE risk, particularly when prosthetic valves or material, residual lesions, or prior IE are present.^2^ Yet procedure-related IE remains uncommon, difficult to attribute to a single exposure, and sometimes recognized only after diagnostic delay. At the catheterization table, the evidentiary challenge is procedure specific. Current guidance emphasizes sterile technique and selective ABP for device or prosthetic implantation rather than routine antibiotics for all clean catheterizations.^3^ A trial powered to detect a modest difference in IE after an invasive exposure, however, would likely require a prohibitively large number of patients.

Against this backdrop, Issa et al^1^ make a useful contribution. In a PHIS analysis of 20,580 pediatric congenital catheterization procedures from 39 pediatric centers between 2016 and 2024, ABP was common but heterogeneous. Overall, 80.5% of procedures included at least one intravenous dose and 49.4% received two or more doses of antibiotics. Among device procedures, 93.9% received at least 1 dose, but 6.1% received none, including 94 transcatheter pulmonary valve replacement (TPVR) cases. Multidose use ranged from 38.8% after patent ductus arteriosus device occlusion to 85.7% after TPVR; conversely, 18% of balloon valvuloplasty cases received at least 1 dose. Early IE within 30 days was rare: only 15 events in 14 patients, an overall incidence of 0.07%. TPVR had the highest procedure-specific incidence (0.27%) and accounted for 53% of early IE events; balloon valvuloplasty, atrial septal defect closure, and patent ductus arteriosus closure had lower rates. The authors found no measurable association between any ABP and early IE (13 of 16,563 with ABP vs 2 of 4,017 without ABP; OR: 1.59; 95% CI: 0.36-7.14; *P* = 0.75).

The central inference should be cautious. This study does not show that ABP is ineffective. With only 15 outcomes, the CI around the exposure estimate is wide enough to include clinically meaningful benefit. Administrative data also cannot fully establish why an antibiotic was administered and whether: 1) timing was optimal; 2) an IE diagnosis was definite; 3) the event presented outside a PHIS hospital; or 4) unmeasured patient and procedural factors drove both antibiotic decisions and IE risk. In this setting, a null association is better interpreted as statistical imprecision rather than therapeutic futility. The paper is most valuable as descriptive epidemiology and as an antimicrobial stewardship map.

This map identifies several actionable signals in our opinion. First, some patients undergoing device implantation apparently received no ABP. For intravascular prosthetic or device procedures, a timed preprocedure dose should be considered routine; not because it can abolish late IE, but because the immediate objective is to reduce procedural inoculation and access-site or device-related infection.^4^ Second, many patients received multiple ABP doses. If prophylaxis is being used for this narrow perioperative objective, the incremental value of postprocedural doses remains uncertain^5^, whereas additional antibiotic exposure can increase the risk of antimicrobial resistance, adverse drug events, and *Clostridioides difficile* infection. Third, for clean catheterizations without device or prosthetic material, ABP should not be routine. Selective use may still be warranted for cases with heightened patient or procedural risk, such as prior IE, complex anatomy/hemodynamics, prolonged instrumentation, or another concurrent infection concern. Centers should therefore standardize procedure-specific protocols, define acceptable exceptions, and audit deviations.

TPVR deserves distinct consideration. TPVR had the highest early IE signal in the PHIS cohort among other transcatheter procedures.^1^ This is not surprising and is due to a number of factors that include: 1) a larger device size; 2) longer procedure time; and 3) frequent placement in a more complex underlying cardiac anatomy. In an international registry of 2,476 TPVR recipients, 182 patients developed IE after a median of 2.7 years; cumulative incidence was 9.5% at 5 years and 16.9% at 8 years, with an annualized incidence of 2.2 per 100 patient-years. *Staphylococcus aureus* and viridans group streptococci together accounted for 56% of cases, and younger age, prior IE, and higher residual right ventricular outflow tract gradient were important risk factors.^6^ This increased risk does not prove that additional perioperative doses prevent early IE, but it does make TPVR categorically different from balloon dilation or a ductal occluder. Systematic reviews further suggest that valve type may influence risk, although confounding remains substantial.^7^ Continued periprocedural ABP for TPVR remains prudent.

The microbiology also deserves more explicit attention. Adult transcatheter aortic valve replacement (TAVR) experience illustrates why “an antibiotic” is not an interchangeable intervention. TAVR-associated IE commonly involves enterococci and staphylococci, and reviews have noted that standard cephalosporin prophylaxis may fail to cover a substantial fraction of early periprocedural pathogens; European discussions have therefore included amoxicillin-clavulanate or ampicillin-sulbactam to improve enterococcal coverage.^8^ TPVR is not TAVR. Congenital patients differ in age, anatomy, prosthesis, access route, and likely skin/access-site microbiology, and available TPVR data show prominent *S aureus* and viridans group streptococci.^6^ Still, the comparison is important: future congenital protocols should align antibiotic selection with expected organisms, local epidemiology, patient risk, and procedure type. PHIS cannot determine whether early IE followed a skin source, oral-source bacteremia, enterococcal infection, or culture-negative disease.

The follow-up window also warrants commentary. A 30-day outcome is defensible for infections plausibly linked to procedural bacteremia or device contamination and potentially modifiable by a single perioperative exposure; the authors also reported similar findings in a 60-day analysis.^1^ Yet device-associated IE can occur later. A single preprocedure antibiotic dose is unlikely to prevent infections months later from dental, skin/soft-tissue, or health care–associated bloodstream infection, but an early procedure-related infection may not be recognized within 30 days, particularly when symptoms are nonspecific, bloodstream infection is intermittent, or prosthetic-valve imaging is challenging. Future studies should therefore evaluate 30-, 90-, and 180-day outcomes while classifying events by microbiology, bloodstream infection, access-site infection, and procedural details.

Where does this leave practice? The best question is not whether every congenital catheterization should receive the same ABP regimen, but which combinations of patient, procedure, prosthetic material, and microbiology create enough preventable risk to justify antibiotic exposure. For now, the middle course is strongest: preserve high reliability in sterile technique and timely preprocedure dosing for device implantation, especially TPVR; avoid routine ABP for clean nondevice catheterizations while defining high-risk exceptions; question routine multidose ABP absent procedure-specific rationale; educate patients and family members that postprocedure fever should prompt immediate evaluation, including blood cultures before antibiotics when clinically appropriate; and build multicenter data sets that include valve type, residual gradients, antibiotic timing and indication, microbiology, access-site infection, and longer follow-up. This approach respects antimicrobial stewardship without ignoring the potentially catastrophic consequences of IE. The paper from Issa et al does not close the debate; it should redirect the field from haphazard practice toward measured, risk-stratified prevention (Figure 1).

**Figure 1:**
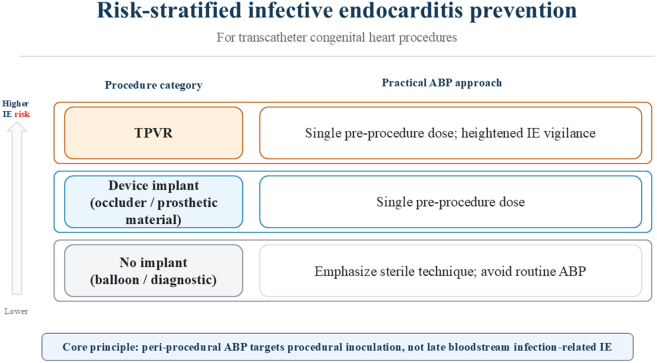
**Risk-Stratified Infective Endocarditis Prevention After Transcatheter Congenital Heart Procedures** ABP = antibiotic prophylaxis; IE = infective endocarditis; TPVR = transcatheter pulmonary valve replacement.

## Funding support and author disclosures

Dr Baddour receives royalty payments for authorship duties from UpToDate, Inc. All other authors have reported that they have no relationships relevant to the contents of this paper to disclose.
